# Changing Epidemiology of Common Cancers in Southern Iran, 2007-2010: A Cross Sectional Study

**DOI:** 10.1371/journal.pone.0155669

**Published:** 2016-05-24

**Authors:** Seyed Masoom Masoompour, Kamran B. Lankarani, Behnam Honarvar, Seyed Hamidreza Tabatabaee, Mohsen Moghadami, Zahra Khosravizadegan

**Affiliations:** 1 Non-Communicable Disease research center, Shiraz University of Medical Sciences, Shiraz, Iran; 2 Health Policy Research Center, Shiraz University of Medical Sciences, Shiraz, Iran; 3 Epidemiology Department, School of Health, Shiraz University of Medical Sciences, Shiraz, Iran; 4 HIV/AIDS Research Center, Shiraz University of Medical Sciences, Shiraz, Iran; 5 Department of Cancer Surveillance, Deputy of Health, Shiraz University of Medical Sciences, Shiraz, Iran; University of South Alabama Mitchell Cancer Institute, UNITED STATES

## Abstract

We have evaluated the ever changing epidemiology of cancers in Fars province, Iran since the re-establishment of Fars cancer registry. Based on the collected data from all related sources in Fars province from 2007–2010 we calculated the cancer age-standardized rates per 100,000 person-years (ASRs). The results are presented as incidence rates of cases by site according to the International Classification of Diseases for Oncology (ICD-O), sex, age, crude rate, and ASRs. In women the total ASR was 41.70 per 100,000 from 1985–1989 which had increased to 55.50 and 95.46 during 1998–2002 and 2007–2010. The incidence of breast cancer in women during 2007–2010 was about two and four times higher than 1998–2002 and 1985–1989. The incidence of colorectal cancer in women during 2007–2010 was about three and five times higher than 1998–2002 and 1985–1989. In men the total ASR was 62.9 per 100,000 in 1985–1989 that increased to 64.50 and 101.48 during 1998–2002 and 2007–2010. Although stomach cancer was the most common cancer among men during 1985–1989 and 1998–2002, but in recent study bladder cancer was the most common cancer among men in Fars province. The incidence of colorectal cancer in men during 2007–2010 was about three times higher than 1998–2002 and 1985–1989. This study shows growing incidence of cancer in southern Iran. The colorectal cancer in both genders had increased and its pattern is similar to western countries. In men, bladder and prostate cancers had a growing rate and the incidences of these cancers in the present study were greater than stomach cancer.

## Introduction

Cancer is the third cause of death and a major health issue in Iran [1]. Despite advanced medical knowledge the main etiology of cancer is not fully known. Assessing the incidence of cancer in various geographic distributions has had an effective role in developing new hypothesis that may not only address the potential etiology of cancer, but also helps to develop novel interventions to prevent cancer or screen the population at risk. The incidence rate within specific region changes through passing of time. While the true increment of cancer in an area could be explained by ever increasing exposure to carcinogens, however, the initial screening programs can show false increases because of early detection of cancer [2]. On the other hand, lifestyle modifications including healthy diet, sanitation (public health) and a well-established screening modalities, can truly reduce cancer incidence rate [3]. The cancer registries provide invaluable information for evaluating cancer occurrence in the context of descriptive epidemiology. The cancer registry in Fars province, southern Iran dates back to 1976 when it was established but its activities were limited to histopathologically confirmed cases without reference to a defined population [4]. The mentioned registry was interrupted because of the revolution and war in the early 1980s. In 1984, the parliament passed a bill mandating the report of all tissues “diagnosed or suspected as cancer” to the Ministry of Health [5]. In 1989, cancer registry in Fars province, southern Iran was resumed and collected the data from all potential resources including pathologic laboratories, radiology clinics, private and teaching hospitals. Monitoring cancer epidemiology changes over time which does not only determines cancer patterns, but also guide planning and evaluation of cancer control programs which helps to set priorities for allocating resources, advance clinical, epidemiologic, and health services research. Although there has been studies on this subject in the Fars province, to the best of our knowledge no study has been published on changing epidemiology of cancers in this region of Iran since the re-establishment of Fars cancer registry program.

## Methods and Materials

### Geography

Fars province, with 122,400 km^2^ area (8% of the total land area of Iran, which is about 1,648,195 km^2^), is located in southern Iran [6]. It is about 5,000 feet above the sea level in a mountainous region of Zagros. Fars province has hot and dry summers, generally mild winters, and a great deal of sunshine throughout the year.

### Fars population

**Fig 1:**
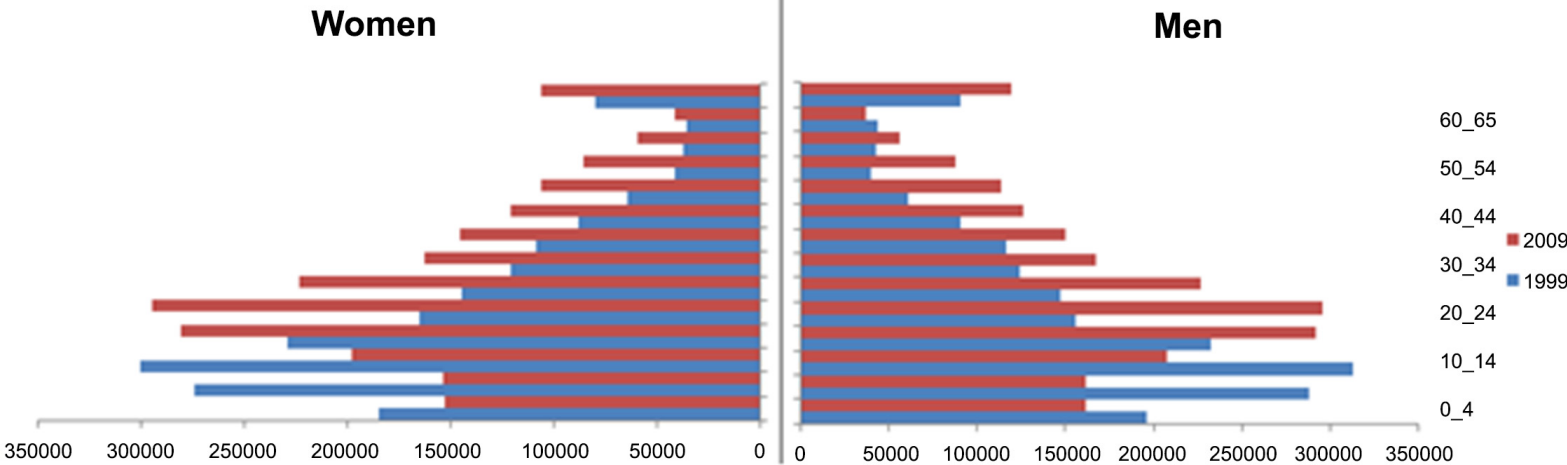
Population pyramid of Fars province, 1999 and 2009. This figure provides population distributions in Fars province including both sexes from 1999 to 2009.

According to the census 1999 and 2009 the total population of Fars province has increased from 3,817,033 (56.5% urban vs. 43.5% rural) to 4,336,878 (61.2% urban vs. 38.8% rural). The mean age (years) of men and women in Fars province has increased from 23.7 and 23.54 in year 1999 to 28.03 and 27.99 in 2009; that indicates aging of the population. Fig 1 shows the population pyramids of Fars province in 1999 and 2009 [7, 8].

### Medical care

Comparing the census of 1999 with 2009 the total number of hospital beds had increased from 5,411 to 6,991 in the entire province [7, 8]. The physician to patient ratio rose from 1:4887 to 1:2681 in the past decade [7, 8].

### Data collection and analysis

In this retrospective study we compared changes in the top ten cancer in our region during three periods; 1985–1989, 1998–2002, and 2007–2010. The raw data of 1985–1989 study was driven from data set that was gathered by Saalabian [9], and we calculated the age-standardized rates per 100,000 person-years (ASRs) for this time period. We published the data on cancer incidence in our region for 1998–2002 [10]. Recently, we conducted a study on cancer incidence in Fars province using data related to 2007 to 2010, (S1 Table). For calculating the cancer ASRs for 2007–2010, we used the data that was gathered from all hospitals, outpatient clinics, pathology laboratories, radiology clinics, and the central death registry office in Fars province cities. We excluded data for those who were nonresident patients who had seeked medical care in Fars province. Considering the ICD-O codes [11], we summarized the data in MS EXCEL (Microsoft, Redmond, WA) software with Persian letterings. The duplicated dada were removed as described in our previous study [10]. The data were computerized using SPSS (Chicago, IL) software, version 13.0. The results are presented as incidence rates of cases by site (ICD-O), sex, age, crude rate, and age-standardized rates per 100,000 person-years (ASRs), using the direct method of standardization to the world population [12]. In order to compare the ASR of 2007–2010 with 1998–2002, we used standardized rate ratio (SRR), as described previously (S2 Table) [12].

### Ethical approval

This study was approved by the ethic committee of Shiraz University of medical Sciences with approval number of EC_P_9398_9098. The ethic committee of Shiraz University of medical Sciences waived informed consent, as this was a retrospective analysis and patients were already consented for research purposes at the time of their recruitment for treatment. Our patients’ records/information was concealed prior to analysis.

## Results

**Fig 2:**
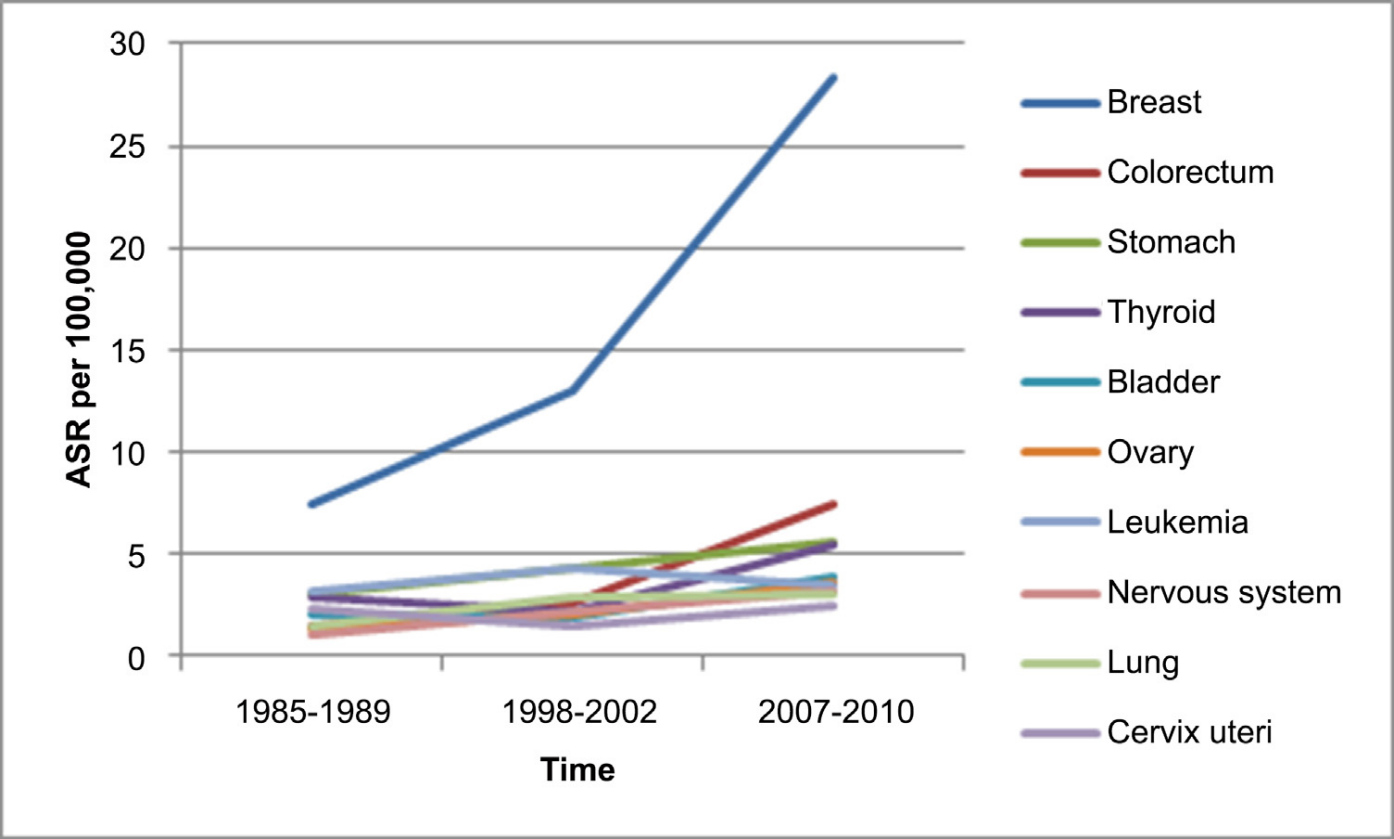
Changing the incidence of top ten cancers in women based on age-standardized rates per 100,000 person-years (ASR) in Fars province, Iran during three time periods. This figure shows changes in the incidence of top ten cancers in women based on age-standardized rates per 100,000 person-years (ASR) in Fars province, Iran during 1985–1989, 1998–2002, and 2007–2010 time periods.

**Fig 3:**
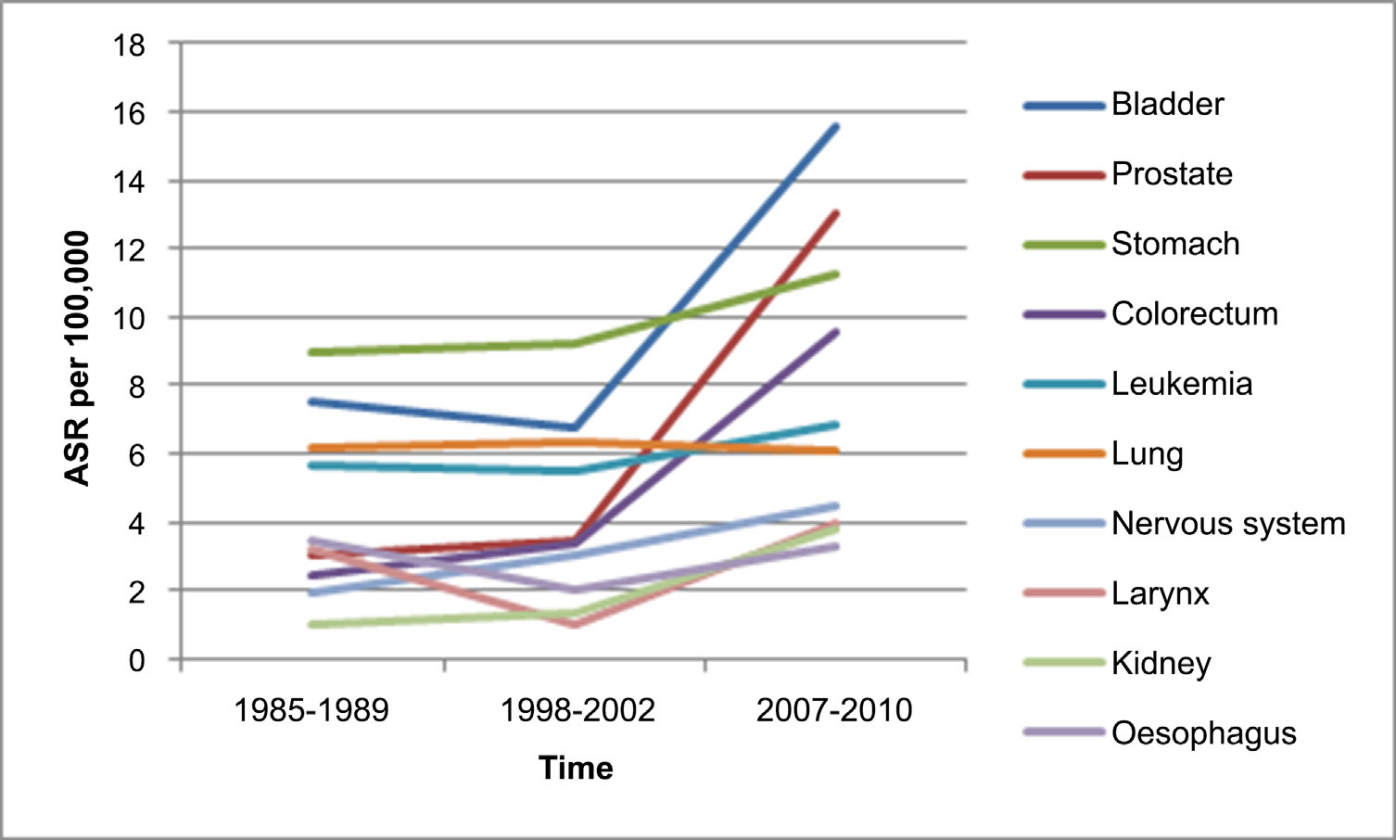
Changing the incidence of top ten cancers in men based on age-standardized rates per 100,000 person-years (ASR) in Fars province, Iran during three time periods. This figure shows changes in the incidence of top ten cancers in men based on age-standardized rates per 100,000 person-years (ASR) in Fars province, Iran during 1985–1989, 1998–2002, and 2007–2010 time period.

The principal cancer sites, ASRs as well as crude rates for both men and women in Fars province, Iran, 2007–2010 are shown in Table 1. Based on calculated ASRs, the 5 most frequent cancers in women were breast (28.37 per 100,000), colon and rectum (7.49 per 100,000), stomach (5.56 per 100,000), thyroid (5.51 per 100,000), and bladder (3.93 per 100,000) (Fig 2); and the 5 most frequent types in men were bladder (15.60 per 100,000), prostate (12.99 per 100,000), stomach (11.21 per 100,000), colon and rectum (9.57 per 100,000), and leukemia (6.82 per 100,000) (Fig 3). The ASR for all cancers in men and women were 101.48 and 95.46 per 100,000 (Table 1).

**Table 1:** Top ten cancers in women and men based on age-standardized rates per 100,000 person-years (ASR) in Fars province, Iran, 2007–2010.

Tumor site	ASR	95% CI	Crude rate	Tumor site	ASR	95% CI	Crude rate
		Women				Men	
Breast	28.37	26.89–29.86	23.35	Bladder	15.60	14.45–16.74	11.65
Colon &rectum	7.49	6.69–8.29	5.64	Prostate	12.99	11.97–14.01	10.17
Stomach	5.56	4.87–6.24	4.18	Stomach	11.21	10.24–12.18	8.52
Thyroid	5.51	4.88–6.14	5.16	Colon &rectum	9.57	8.67–10.46	7.21
Bladder	3.93	3.35–4.51	2.90	Leukemia	6.82	6.11–7.53	5.73
Ovary	3.64	3.1–4.17	3.02	Lung	6.09	5.39–6.8	4.71
Leukemia	3.49	2.97–4.01	2.98	Nervous system	4.44	3.87–5.01	3.90
Nervous system	3.26	2.73–3.78	2.44	Larynx	3.93	3.34–4.52	2.78
Lung	3.04	2.53–3.56	2.25	Kidney	3.81	3.24–4.38	2.84
Cervix uteri	2.55	2.09–3.01	1.97	Esophagus	3.25	2.72–3.78	2.39
Total	95.46	92.69–98.22	77.52	Total	101.48	98.61–104.35	79.76

Comparing the results of three time periods (1985–1989, 1998–2002, and 2007–2010) are shown in Tables 2 and 3 for women and men. The total trend of ASR of cancer for both sexes in Fars province is incremental. In women the total ASR was 41.70 per 100,000 in 1985–1989 that increased to 55.50 and 95.46 during 1998–2002 and 2007–2010 (Table 2). Breast cancer was the most common cancer among women during all time periods. The incidence of breast cancer in women during 2007–2010 was about two and four times higher than 1998–2002 and 1985–1989. The incidence of colorectal cancer in women during 2007–2010 was about three and five times higher than 1998–2002 and 1985–1989.

**Table 2:** Top ten cancers in women based on age-standardized rates per 100,000 person-years (ASR) and standardized rate ratio (SRR) in Fars province comparing to previous studies in Fars, GLOBOCAN 2008.

Tumor sites	Fars 1985–89	Fars 1998–02	Fars2007-10	SRR* (95%)	GLOBOCAN^13^2008
Breast	7.40	13.00	28.37	2.2(1.9–2.5)	18.40
Colon &rectum	1.50	2.60	7.49	2.9(2.4–3.5)	6.40
Stomach	3.00	4.40	5.56	1.3(1.1–1.5)	9.00
Thyroid	2.90	2.20	5.51	2.5(2.2–2.9)	2.30
Bladder	2.00	1.90	3.93	2.1(1.8–2.4)	2.60
Ovary	1.40	2.00	3.64	1.8(1.6–2.1)	3.10
Leukemia	3.20	4.40	3.49	0.8(0.7–0.9)	4.00
nervous system	1.10	2.20	3.26	1.5(1.3–1.8)	2.10
Lung	1.50	2.90	3.04	1(0.9–1.2)	3.50
Cervix uteri	2.30	1.50	2.55	1.7(1.5–2)	2.20
Total	41.70	55.50	95.46	1.7(1.5–2)	87.50

* SRR: **Standardized Rate Ratio, ASR2007-10/ASR 1998–02**

**Table 3:** Top ten cancers in men based on age-standardized rates per 100,000 person-years (ASR) and standardized rate ratio (SRR) in Fars province comparing to previous studies in Fars, GLOBOCAN 2008.

Tumor sites	Fars 1985–89	Fars1998-02	Fars2007-10	SRR* (95%)	GLOBOCAN^13^2008
Bladder	7.50	6.80	15.60	2.3(2–2.7)	11.20
Prostate	3.00	3.50	12.99	3.7(3.1–4.4)	11.60
Stomach	9.00	9.20	11.21	1.2(1–1.4)	21.90
Colon &rectum	2.40	3.40	9.57	2.8(2.3–3.4)	8.70
Leukemia	5.70	5.50	6.82	1.2(1.1–1.3)	7.70
Lung	6.20	6.30	6.09	1(0.9–1.1)	9.10
nervous system	1.90	3.00	4.44	1.5(1.3–1.7)	3.60
Larynx	3.20	1.00	3.93	3.9(3.3–4.7)	3.50
Kidney	0.97	1.30	3.81	2.9(2.4–3.5)	2.70
Esophagus	3.50	2.00	3.25	1.6(1.4–1.9)	7.40
Total	62.90	64.50	101.48	1.6(1.4–1.8)	126.10

* SRR: **Standardized Rate Ratio, ASR2007-10/ASR 1998–02**

In men the total ASR was 62.9 per 100000 in 1985–1989 that increased to 64.50 and 101.48 during 1998–2002 and 2007–2010 (Table 3). Although stomach cancer was the most common cancer among men during 1985–1989 and 1998–2002, in a recent study bladder cancer was the most common cancer among men in Fars province. The incidence of bladder cancer in men during 2007–2010 was about two times higher than 1998–2002 and 1985–1989. The incidence of colorectal cancer in men during 2007–2010 was about three times higher than 1998–2002 and 1985–1989.

## Discussion

This study shows the growing incidence rate of cancer in Fars province. Compared with 1998–2002, the incidence of breast, colorectal, and stomach cancers in women grew much more during 2007–2010. Compared with 1998–2002, the incidence of bladder, prostate and colorectal cancers in men grew much more during 2007–2010. The growing trend of cancer in Fars province follows the overall trend of cancer in the world and especially in developing countries [13]. The growing pattern of cancer compared with previous study in Fars could be multi factorial. Aging of population could be another issue. On average Fars population got 5 years older during a decade [7, 8]. Starting and intensification of new screening programs occurred during the same time period; Mammography for women and prostate exam for men during men's health day. Access to new diagnostic modalities such as new version of computerized tomography might also affect the higher diagnosis rate. Improved sanitation and decreased infectious diseases along with increasing population age, changing life styles including; more use of unsanitary fast foods, more food consumption, increasing transplantation operation and subsequently higher use of cytotoxic drug might all had contributed to this issue. Starting and establishing the population based cancer registries that occurred following parliament mandate to report all tissues “diagnosed or suspected of cancer” [5], and finally more applying information technology in the medical field including cancer could have played an important role.

Below we compare the incidence rates for top ten cancers in decreasing order from the present study with the rates from the previous provincial cancer registry. The implications of notable changes in incidence rates are also discussed.

### Breast cancer

The incidence trend of breast cancer, as the most common cancer in women is growing and has risen by two fold each decade since 1980s [10]. Although this increase could be mainly due to the increased use of a screening program, but other factors such as decrease in protective factor like decrease in birth rate and breast-feeding rate should also be considered [14]. The breast cancer incidence was higher than other provinces [15–18] and Globocan 2008 (28.37 per 100,000 vs. 18.4 per 100,000) [13].

### Bladder cancer

In the present study bladder cancer is the most common cancer in men (15.6 per 100,000) and fifth most common in women (3.93 per 100,000). Men to women ratio were 3.97:1 and were similar to Salehi’s study [19] and other previous studies in Iran [20]. The incidence of bladder cancer has shown an incremental trend comparing to previous study [10]. The incremental trend of bladder cancer cannot solely be explained by improvement in diagnostic methods, as bladder cancer can be diagnosed by cystoscopy and biopsy for many years [21]. Possible explanation for the increase in ASR for bladder cancer may be due to increase in number of smokers and exposure to other industrial carcinogens and population [22]. There are reports of long term oncogenic effects of opium usage which may have contributed to this increasing trend [23].

### Prostate cancer

Prostate cancer with 12.99 per 100,000 incidences is the second most common cancer in men in Fars province. It had risen four times when compared to a previous study in Fars [10]. This increase in incidence of prostate cancer cannot not only due to population based screening program for prostate cancer but also due to aging population and increase in rate of smokers [24, 25].

### Stomach cancer

In this study stomach cancer is the third most common cancer in both genders. Although the incidence of stomach cancer has relatively increased from 4.4 to 5.56 per 100,000 in women and 9.2 to 11.21 per 100,000 in men comparing with older Fars study [10], but these incidences are much lower than those reported in GLOBOCAN 2008 [13], and the northern provinces of Iran [15–18]. The lower rate of stomach cancer in our province could be due to lower H. pylori infection [26], and less consumption of smoked or preserved foods in Fars population.

The proximal gastric cancer compared to distal gastric cancer has a negative correlation with H.pylori infection [27]. Since no distinction has been made between these two subtypes, thus one cannot be sure of absolute increase in the number of gastric cancers of its subtypes. It is postulated that this might be due to more proximal gastric cancers. Some of these cancers were previously mistaken for distal esophageal adenocarcinoma and better classification might have had an effect on this increase of ASR for gastric cancer.

### Colorectal cancer

Colorectal cancer is the second most common cancer in women and fourth in men. Comparing with previous study [10] the incidence of colorectal cancer had risen by three fold. Changing dietary habit, consumption of fast food and low fiber diet [14], can be associated with increased colonoscopy facility could explain the greater incidence of colorectal cancer in our region.

### Leukemia

The incidence of leukemia in men was nearly twice as much as women (6.82 vs. 3.49 per 100,000). The main reason was that men were more exposed to risk factors such as; smoking and occupational and environmental hazards like chemical agents, pesticides and radiation [28, 29].

### Lung and larynx cancer

The incidence of lung cancer in both genders did not show any significant changes compared with 1998–2002 study [10]. One explanation is that in previous study some misclassification of metastatic lung cancer as primary lung cancer had occurred, however, recently with progress in radiologic technology and diagnostic laboratory (Immunohistochemistry) this misclassification has been rectified. On the other hands, based on previous study more than 80% of lung cancer had been verified microscopically which means in our cancer registry system, patients with lung cancer could have been labeled as pneumonia or other impressions [10].

### Thyroid cancer

Thyroid cancer is the fourth most common amongst women (5.51 per 100,000), while in men this incidence was 1.64 per 100,000. A woman to men ratio in 1998–2002 was 2, but this ratio had risen to 3.35 in a recent study. Both the increasing use of diagnostic technologies and a true increase in thyroid cancer incidence could have played a role in this issue. The women in Fars body mass index (BMI) had increased more than men [30], and obesity has a strongest link to thyroid cancer risk among other risk factors [31].

### Nervous system cancer

Nervous system cancer showed an increased incidence rate among both genders during 2007–2010 compared with 1998–2002. Men predominance pattern of nervous system cancer was similar in the present study compared with older study in Fars province [32]. Whilst high dose ionizing radiation is an established risk factor for this group of tumors [33], imaging modalities for diagnosis of nervous system cancer was more available during 2007–2010 compared to 1998–2002.

### Esophageal cancer

In Fars province, esophageal cancer was much lower than north of Iran, nevertheless the incidence of esophageal cancer had increased during 2007–2010 period (Women 2.04 vs. 1.4 and men 3.25 vs. 2 per 100,000). There are two major types of esophageal cancer: squamous cell carcinoma (SCC) and adenocarcinoma (AC). Most of esophageal cancers in northern Iran were SCC [34]. The risk factors for these two subtypes are very different. Exposure to nitrosamines, polyaromatic hydrocarbons, smoked food, hot beverages and opium are considered as risk factors for SCC, while AC is mostly related to chronic gastroesophageal reflux disease [35]. However, the data that distinct these two subtypes is not available. But there has been a huge increase in incidence of gastroesophageal reflux disease in recent years in Iran [36]. This may have contributed to some increase in ASR of esophageal cancer.

### Ovarian and cervical cancer

Both ovarian (3.64 vs. 2 per 100,000) and cervical (2.55 vs. 1.5 per 100,000) cancer increased during 2007–2010. Part of this increase was probably related to better diagnosis due to more screening and diagnostic modes. Screening for cervical cancer was part of the basic health service in public health facilities. A diagnostic mode for ovarian cancer is mainly sonography which is now more widely available in the province. Older age of the studied population might have also contributed to this issue.

### Limitations

Considering that the methodology of our study was retrospective, this study has several potential limitations. Our cancer registry only recorded cancer cases instead of patients, so in situation that a patient had more than one primary cancer, the same person was counted more than once [37]. Although we did our best to omit duplicated reports, but possible duplication may have occurred equally across all types of cancers and in both genders, consequently the possibility of occurrence of systematic errors in our analysis would be highly unlikely. Since our cancer registry system emphasis on documentation of cancer cases using pathologic and microscopic verification, our findings could have underestimate the incidence of cancer in our region.

## Conclusions

This study shows growing incidence rate of cancer in Fars province, southern Iran. The colorectal cancer in both genders has increased and its pattern was similar to western countries. In men, bladder and prostate cancers had an increasing rate and their incidence in the present study was greater than stomach cancer. However, breast cancer is the most common type of cancer in women as it was shown in previous study.

## Supporting Information

S1 Table Dataset of the cancer cases in Fars province, Iran, 2007–2010. (XLS)

S2 Table Calculation of standardized rate ratio (SRR) between ASR 2007–10 and ASR 1998–02. (XLSX)

## References

[1] Naghavi M, Abolhassani F, Pourmalek F, Lakeh M, Jafari N, Vaseghi S, et al The burden of disease and injury in Iran 2003. Popul Health Metr. 2009; 7: 9 10.1186/1478-7954-7-9 19527516 PMC2711041

[2] Jemal A, Center MM, Ward E, Thun MJ. Cancer occurrence. Methods Mol Biol. 2009;471:3–29. 10.1007/978-1-59745-416-2_1 19109772

[3] Belpomme D, Irigaray P, Sasco AJ, Newby JA, Howard V, Clapp R, et al The growing incidence of cancer: role of lifestyle and screening detection. Int J Oncol. 2007; 30, 1037–49. 17390005 10.3892/ijo.30.5.1037

[4] Etemadi A, Sadjadi A, Semnani S, Nouraie SM, Khademi H, Bahadori M. Cancer registry in Iran: a brief overview. Arch Iran Med. 2008; 11: 577–580. 18759534

[5] Islamic Republic of Iran Parliamentary Laws. Mandatory Registration of Cancer. Available: URL: http://lawoffice.mohme.gov.ir. [Persian]. Accessed 7 April 2008.

[6] Wikipedia. Fars Province. Available: http://en.wikipedia.org/wiki/Fars_Province. Accessed 10 April 2011.

[7] Statistical Center of Iran. Iran statistical yearbook Tehran, Iran: Statistical Center of Iran, Press; 1999.

[8] Statistical Center of Iran. Iran statistical yearbook Tehran, Iran: Statistical Center of Iran, Press; 2009.

[9] Saalabian MJ. Registry of neoplastic diseases Fars province 1985–89. Shiraz, Iran: Shiraz University of Medical Sciences, Press 1990.

[10] Masoompour SM, Yarmohammadi H, Rezaianzadeh A, Lankarani KB. Cancer incidence in southern Iran, 1998–2002: Results of population-based cancer registry. Cancer Epidemiol, 2011;35: 42–7. 10.1016/j.canep.2011.05.018 21840285

[11] Fritz PA, Percy C, Jack A. International classification of diseases for oncology, 3rd ed., Geneva: WHO, Press; 2000.

[12] Jensen OM, Parkin DM, Maclennau R, Mair CS, RG S. Cancer registration: principles and methods, 1st ed., Lyon: IARC, Press; 1991.

[13] Ferlay J, Shin HR, Bray F, Forman D, Mathers C, Parkin DM. GLOBOCAN 2008 Cancer incidence and mortality worldwide: IARC cancerbase no. 10 [Internet]. Lyon, France: International Agency for Research on Cancer, 2010.

[14] Boffetta P, Parkin DM. Cancer in developing countries. CA Cancer J Clin 1994;44:81–90. 8124607 10.3322/canjclin.44.2.81

[15] Talaiezadeh A, Tabesh H, Sattari A, Ebrahimi S. Cancer incidence in southwest of Iran: first report from khuzestan population-based cancer registry, 2002–2009. Asian Pac J Cancer Prev. 2013;14(12):7517–22. 24460327 10.7314/apjcp.2013.14.12.7517

[16] Somi MH, Farhang S, Mirinezhad SK, Naghashi S, Seif-Farshad M, Golzari M. Cancer in East Azerbaijan, Iran: results of a population-based cancer registry. Asian Pac J Cancer Prev. 2008 Apr-Jun;9(2):327–30. 18712985

[17] Roshandel G, Sadjadi A, Aarabi M, Keshtkar A, Sedaghat SM, Nouraie SM, et al Cancer incidence in Golestan Province: report of an ongoing population-based cancer registry in Iran between 2004 and 2008. Arch Iran Med. 2012 4;15(4):196–200. doi: 012154/AIM.004 22424034

[18] Babaei M, Jaafarzadeh H, Sadjadi AR, Samadi F, Yazdanbod A, Fallah M, et al Cancer incidence and mortality in Ardabil: report of an ongoing Population-Based Cancer Registry in Iran, 2004–2006. Iran J Public Health. 2009; 38: 35–45.

[19] Salehi A, Khezri AA, Malekmakan L, Aminsharifi A. Epidemiologic status of bladder cancer in Shiraz, southern Iran. Asian Pacific J Cancer Prev. 2011;12(5):1323–7. 21875290

[20] Yavari P, Sadrolhefazi B, Mohagheghi MA, Mehrazin R. A descriptive retrospective study of bladder cancer at a hospital in Iran (1973–2003). Asian Pac J Cancer Prev. 2009 Oct-Dec;10(4):681–4. 19827894

[21] Madeb R, Messing EM. Gender, racial and age differences in bladder cancer incidence and mortality. Urol Oncol. 2004 Mar-Apr;22(2):86–92. 15082003 10.1016/S1078-1439(03)00139-X

[22] Costa LAV, Wroclawski ML, Machado MT, Pompeo ACL, Wroclawski ER. Non-occupational risk factors for bladder. Einstein. 2008; 6(4): 507–10.

[23] Aliasgari MA, Kaviani A, Gachkar L, Hosseini-Nassab SR. Is bladder cancer more common among opium addicts? Urol J. 2004 Fall;1(4):253–5. 17914701

[24] Mousavi SM. Toward prostate cancer early detection in Iran. Asian Pac J Cancer Prev. 2009 Jul-Sep;10(3):413–8. 19640184

[25] Islami F, Moreira DM, Boffetta P, Freedland SJ. A Systematic Review and Meta-analysis of Tobacco Use and Prostate Cancer Mortality and Incidence in Prospective Cohort Studies.Eur Urol. 2014 12;66(6):1054–64. 10.1016/j.eururo.2014.08.059 25242554 PMC4566150

[26] Farshad S, Japoni A, Alborzi A, Zarenezhad M, Ranjbar R. Changing prevalence of Helicobacter pylori in south of Iran. Iranian J Clin Infect Dis. 2010;5:65–9.

[27] Kuipers EJ. Review article: exploring the link between Helicobacter pylori and gastric cancer. Aliment Pharmacol Ther. 1999 3;13 Suppl 1:3–11. 10209681 10.1046/j.1365-2036.1999.00002.x

[28] Groves FD, Linet MS, Devesa SS. Epidemiology of human leukemia. Curr Opin Hematol. 1994 7;1(4):321–6. 9371299

[29] Maryam Z, Sajad A, Maral N, Zahra L, Sima P, Zeinab A, et al Relationship between exposure to pesticides and occurrence of acute leukemia in Iran. Asian Pac J Cancer Prev. 2015;16(1):239–44. 25640359 10.7314/apjcp.2015.16.1.239

[30] Pishdad GR. Overweight and obesity in adults aged 20–74 in southern Iran. Int J Obes Relat Metab Disord. 1996 10;20(10):963–5. 8910103

[31] Peterson E, De P, Nuttall R. BMI, Diet and Female Reproductive Factors as Risks for Thyroid Cancer: A Systematic Review. PLoS One. 2012; 7(1): e29177 10.1371/journal.pone.0029177 22276106 PMC3261873

[32] Jazayeri SB, Rahimi-Movaghar V, Shokraneh F, Saadat S, Ramezani R. Epidemiology of primary CNS tumors in Iran: a systematic review. Asian Pac J Cancer Prev. 2013;14(6):3979–85. 23886218 10.7314/apjcp.2013.14.6.3979

[33] McKinney PA. Central nervous system tumors in children: epidemiology and risk factors. Bioelectromagnetics. 2005;Suppl 7:S60–8. 16142778 10.1002/bem.20149

[34] Lankarani KB, Mowla A, Asadian F, Tabei SZ, Panjehshahin MR. Changing epidemiology of esophageal cancer in Fars province, Iran. Iran J Med Sci. 2002;1:10–4.

[35] Jakszyn P, Gonzalez CA. Nitrosamine and related food intake and gastric and oesophageal cancer risk: A systematic review of the epidemiological evidence. World J Gastroenterol. 2006;12(27):4296–303. 16865769 10.3748/wjg.v12.i27.4296 PMC4087738

[36] Pourhoseingholi A, Pourhoseingholi MA, Moghimi-Dehkordi B, Barzegar F, Safaee A, Vahedi M, et al Epidemiological features of gastro-esophageal reflux disease in Iran based on general population. Gastroenterol Hepatol Bed Bench. 2012 Winter;5(1):54–9. 24834199 PMC4017448

[37] Izquierdo JN, Schoenbach VJ. The potential and limitations of data from population-based state cancer registries. Am J Public Health 2000;90: 695–8. 10800415 10.2105/ajph.90.5.695 PMC1446235

